# Comparative analysis of carbon footprint between conventional smallholder operation and innovative largescale farming of urban agriculture in Beijing, China

**DOI:** 10.7717/peerj.11632

**Published:** 2021-06-29

**Authors:** Yingjie Hu, Jin Sun, Ji Zheng

**Affiliations:** 1 College of City Construction, Jiangxi Normal University, Nanchang, Jiangxi, China; 2 College of Surveying and Geo-informatics, North China University of Water Resources and Electric Power, Zhengzhou, Henan, China; 3 Department of Urban Planning and Design, The University of Hong Kong, Pokfulam, Hong Kong SAR, China

**Keywords:** Life cycle assessment, Carbon footprint, Conventional smallholder operation, Home-delivery vegetable, Pick-your-own fruit, Beijing

## Abstract

The sustainable development of agriculture is one of the key issues of ensuring food security and mitigating climate change. Since innovative large-scale agriculture is gaining popularity in cities in China, where the agricultural landscape is dominated by conventional smallholder farming, it is necessary to investigate the difference in carbon emissions between conventional smallholder operation and innovative largescale agriculture. This study evaluated the carbon footprint (CF) of conventional and innovative urban agriculture in Beijing using the cradle-to-consumption Life Cycle Assessment (LCA). Two modes of greenhouse vegetable and fruit production were analyzed and compared respectively: conventional smallholder operated vegetable farms that sell in local markets versus largescale home-delivery agriculture (HDA) that deliver vegetables to consumers’ home directly, conventional smallholder operated fruit farms that sell in farm shops versus largescale pick-your-own (PYO) initiatives. Results showed that HDA and PYO can reduce CF per area in on-farm cultivation compared to smallholder operation, while may bring an increase in CF per product weight unit and the gap was wider if the supply chain was considered. This is mainly because innovative large-scale farming consumes fewer agricultural inputs (e.g., fertilizer, pesticides) and obtains lower yields than conventional smallholder operations. Plastic materials with high carbon emission, fossil energy dependence and transportation efficiency are CF hotspots of both modes and therefore can be prioritized and targeted for carbon reduction adjustment. The results of this work further advance understanding of how innovative largescale agriculture and conventional smallholder operation compare and which particular inputs and activities should be prioritized to effectively reduce the CF in China during agricultural transformation.

## Introduction

Ensuring food security and mitigating climate change are both major pillars of sustainable human development. Agriculture occupies 37.1% of the world’s land area, provides sufficient food for 5662.1 million people, and emits 5326.0 million tonnes (Mt CO_2_-eq) of greenhouse gas (GHG) in 2016 (FAOSTAT, 2019). According to the estimation from IPCC (2014), agriculture, forestry and other land use is responsible for about 25% of the total global annual CO_2_ emissions, and the proportion will be higher when fossil fuel CO_2_ emissions from agricultural use in machinery, such as tractors, irrigation pumps, etc. are included (Ceschia et al., 2010). As one of the most populous countries in the world, China is the leading agricultural carbon emitter and the carbon emission from its agriculture sector reached almost 691.23 million tonnes (Mt CO_2_-eq) in 2016, accounting for approximately 13.0% of the global agricultural carbon emission (FAOSTAT, 2019). Against the backdrop of population growth and climate change, the agriculture sector has increased its environmental and political relevance in recent years. Government policy is attempting to reduce carbon emissions in agriculture through establishing advisory bodies such as the Low-carbon Agriculture Committee, and implementing action programs (SCPRC, 2016) for more sustainable agriculture.

As one of inescapable challenges in tackling agricultural GHG emissions, urbanization has been rapidly expanding worldwide and is expected to go further over the coming decades. About 53.6% (3.88 billion) of the global population now lives in urban areas and this figure is projected to be at approximately 66.4% (6.34 billion) by 2050 (UNDSEA, 2015). Agriculture in cities plays an important role in food production and food security, together with its health and nutrition aspects (Gerster-Bentaya, 2015), forming a key component of the global sustainable food system (Maxwell, 2003). It also provides recreational (recreational routes, food buying on the farm, visiting facilities, etc.) or educational opportunities for citizens (bringing youth in contact with crops, teaching about agronomy or ecology, etc.) (De Zeeuw, 2003). In addition to the benefits above, urban agriculture may also contribute to the improvement or the deterioration of urban environments, and it leads to the increase of carbon footprint if not planned and practiced wisely and in an environmentally friendly way (Goldstein et al., 2016; Mok et al., 2014). Along with the ongoing growing population agglomeration, cites are facing unprecedented challenges of climate change and food security, and urban agriculture has gained great attention of environmentalists and urban planners (Kulak, Graves & Chatterton, 2013).

Smallholder farming that each operate a few hectares of land and a few types of crops dominates the agricultural landscape in China, due to the large population and severe scarcity of arable land (Cui et al., 2018). Since the Reform and Opening-Up, China has been experiencing an unprecedented and remarkable urbanization process (Normile, 2008) and a series of innovative operation modes emerged in cities to meet the enhancing demand of residents for fresh agricultural products and recreational farming experience (Yang et al., 2016). Compared with the conventional smallholder operation, these innovative agriculture modes were usually operated by enterprises or agricultural cooperatives, characterized by large-scale in cultivated area and diversity in crop types (Table 1). Most innovative large-scale agriculture directly sell the fresh product to the consumers (e.g., home-delivery agriculture, community-supported farm) or attract the consumers to come to the farm (e.g., pick-your-own operation, sightseeing garden). The home-delivery vegetable garden and pick-your-own fruit ranch are the most extended and representative innovative largescale agriculture modes, which bring together consumers and farmers within the sphere of direct contact and transparency. The innovative large-scale operation is generally regarded as a potential option for reducing agricultural carbon emissions by improving resource use efficiency and reducing material and energy consumption (Zhu et al., 2018). Within the growing trend of the shorting of supply chain and direct sale, the conventional smallholders in urban areas also explore direct sale channels (e.g., farm shop, local market) to reduce or eliminate the intermediaries of agricultural products from field to fork (Hu et al., 2019). Therefore, it is essential to estimate and compare the carbon emission of the conventional smallholder operation and innovative large-scale agriculture, contributing to the low-carbon development of China in agricultural transformation.

**Table 1:** Description of the conventional and innovative agricultural modes in China.

	Conventional modes	Innovative modes
Operator	Smallholder	Enterprises or agricultural cooperatives
Cultivated area	Relatively small	Relatively large
Labor	Relatively less, most rely on household labor force, occasionally need temporary employment	Relatively more, most have long-term employees
Crop types	Usually only one or a few	Diversified
Supply chain	1) conventional supply chain with multi-intermediaries (e.g., cooperative, wholesaler, retailer, etc.) ***2)direct sale to consumers in local markets without intermediaries***	Direct sale without intermediaries: ***1) home-delivery*** ***2) pick-your-own*** 3) agricultural sightseeing gardens 4) community-supported agriculture …

The carbon emission of urban agriculture is significantly affected by the infrastructure, field management, and distribution. The heated greenhouse production system generally resulted in higher carbon emission than unheated greenhouse and open-field cultivation systems (Ntinas et al., 2017; Soode-Schimonsky, Richter & Blaschke, 2017). The energy used for heating, e.g., electricity, fossil fuels, renewable biofuels, geothermal or solar energy (Boulard et al., 2011; Torrellas et al., 2012b), and the insulation techniques (Fox, Adriaanse & Stacey, 2019; Vadiee & Martin, 2012) varied the carbon emission in heated greenhouse production. The material inputs, including the fertilizer application rate and the consideration of mineral fertilizer vs. manure, also greatly affect the carbon emission results. In addition to the on-farm production process, the difference in supply chains cause the variation in the carbon emissions as well. The shortening of supply chain was generally proposed to be beneficial for the carbon emission reduction compared with long-distance transported food (Page, Ridoutt & Bellotti, 2012; Stoessel et al., 2012). The way of transportation also had an impact on the carbon emissions (Coley, Howard & Winter, 2009). Therefore, the assessment of agricultural carbon emission should consider the entire life cycle, including the agricultural production and the subsequent stages of supply chains, to provide insights for developing improvements (Theurl et al., 2014).

In terms of assessment method, the Life Cycle Assessment (LCA) is a methodological framework for estimating and assessing the environmental impacts attributable to the life cycle of a product, service or activity (Guinée et al., 2011; Lee, O’Callaghan & Allen, 1995; Rebitzer et al., 2004). Whilst LCA has most initially been used in industry as a tool for process selection and optimization (Azapagic, 1999), it has also been extensively applied in the evaluation of the environmental impacts associated with agricultural systems and food products (Lopes, Medeiros & Kiperstok, 2018; Parajuli, Thoma & Matlock, 2019). The LCA comprises four steps: (1) goal definition and scoping, (2) life cycle inventory analysis, (3) impact assessment, and (4) interpretation (Rebitzer et al., 2004; Shiina et al., 2011). It allows objective quantification and hotspot identification of the environmental impact in terms of a series of indices such as carbon footprint (CF) (Torrellas et al., 2012a). It also can identify differences in the environmental impacts among different systems with equivalent functions (Blengini & Busto, 2009; Cellura, Longo & Mistretta, 2012). Therefore, the evaluation and comparison of the CF of different urban agriculture modes could be conducted by LCA analysis.

Regarding the innovative urban agriculture modes, some precedents are worth considering. Pérez-Neira & Grollmus-Venegas (2018) evaluated the carbon footprint of peri-urban horticulture in Spain through a cradle-to-consumption LCA approach. They studied and compared the two conventional farms that sell their output through a conventional local distribution system, and a community-supported agricultural initiative that sells its organic vegetables directly to the consumers (Pérez-Neira & Grollmus-Venegas, 2018). Another study emphasized the food-related GHG emission reduction potential that could be achieved through urban agriculture in the London Borough of Sutton, by comparing the GHG emissions of the urban community farm and the conventional food supply system (Kulak, Graves & Chatterton, 2013). Sanyé-Mengual et al. (2015) conducted an environmental LCA of rooftop greenhouse implementation in Barcelona, Spain, and found this new form of urban agriculture embody higher environmental burdens, including carbon emission, than conventional multi-tunnel greenhouse. Regarding geographical representation, previous research focused mostly on Europe and only a few other regions. Most studies of agricultural carbon emissions in China focused on grain crops production from regional scale (Xu & Lan, 2017; Yan et al., 2015; Yao et al., 2017) and the research on different agriculture modes in cities is deficient. Moreover, a few prior researches about the carbon emission of urban vegetable in China only took the on-farm cultivation into account without considering the post-farm phase (He et al., 2016; Jia, Ma & Xiong, 2012). Within the above analytical framework, it is essential for China to make life cycle environmental evaluation of vegetable and fruit production in different urban agriculture modes, especially to unveiling the diversity of the on-farm production managements and off-farm distribution chains.

Consequently, the primary objective of this study is to evaluate and compare the CF of conventional smallholder operation and innovative largescale agriculture in Beijing taking into consideration the differences between field management and supply chains. The second objective is to identify the CF hotspots in the life cycle of vegetable and fruit production that might be reduced by management changes. For the purposes above, a cradle-to-consumption LCA method was applied on four different types of farms: (1) conventional smallholder operated vegetable farm that directly sell to consumers in local markets; (2) innovative large-scale home-delivery agriculture (HDA) that deliver their vegetables to the consumers’ home directly; (3) conventional smallholder operated fruit farm that directly sell to consumers right in farm shops; (4) innovative large-scale “pick your own” agriculture (PYO) in which customers pick the fruits off by themselves. The analysis in this paper provides contextualized scientific information that could contribute to the urban agricultural projects designing and policy-making to achieve the strategic objective of carbon emission reduction and sustainable development.

## Materials & Methods

### Case description and data collection

The farms were selected based on the representativeness of a) two different production modes (conventional small-scale household-operated versus large-scale farming with employees) and b) different direct supply chains (vegetable sale in local markets versus home-delivery, fruit sale in farm shops versus “pick-your-own”). To enable an unbiased comparison, all the chosen farms used the same type of single-sloped plastic covered greenhouse without heating or CO_2_ enriching system.

VC represented conventional smallholder operations that cultivate small-scale vegetable farms with narrow kinds of crops and sell to consumers directly in nearby local markets.

VN corresponded to innovative home-delivery agriculture (HDA) initiatives that cultivate relatively larger farm with a variety of vegetables and directly deliver to consumers’ home door by door. The consumers of these HDA initiatives annually pre-paid for regular vegetable delivery. A serving (5 kg) of vegetables was delivered each time and the frequency was usually once or twice a week, and a few customers choose deliveries three times a week.

FC represented conventional smallholder operations that cultivate small-scale farms with only one or two kinds of fruit and directly sell to consumers right in the farm shop.

FN corresponded to innovative “pick-your-own” (PYO) initiatives that cultivate relatively large area of fruits. The consumers of these PYO initiatives pick fruits off the plants by themselves. Farmers in some PYO initiatives may pick and package the fruits and make them available for customer selection to reduce wastage on the part of customers who may be unaware of how to select the product.

**Figure 1:**
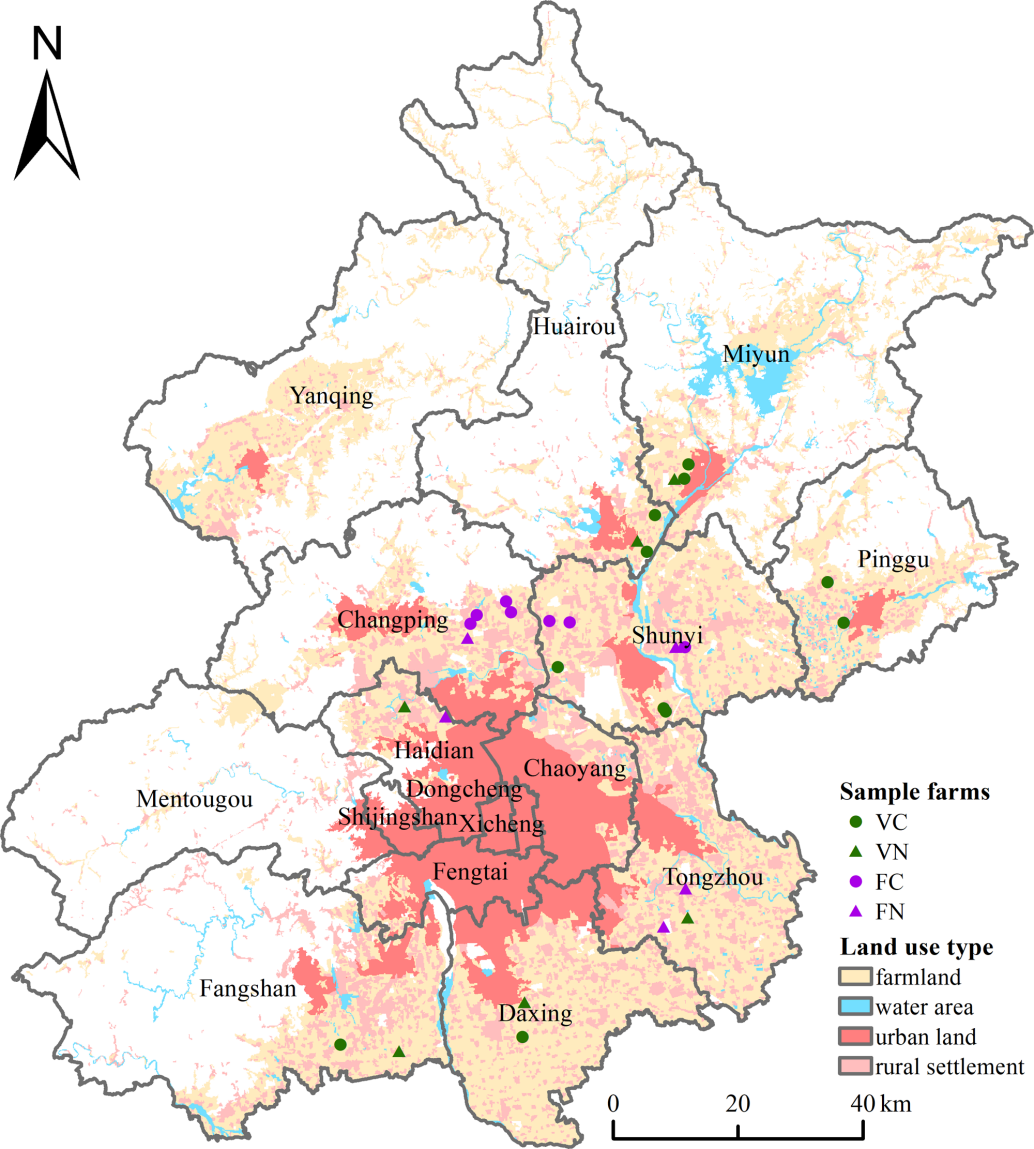
Location of the sample farms in Beijing.

The information required to make the environmental estimates was collected through face-to-face questionnaires in the field survey during July 18-29, 2016. Detailed person-to-person interviews with the individual peasants and largescale farm managers were undertaken to understand how the farms were managed, in particular, regarding cropping patterns, farming techniques and machinery, the input of materials, the consumption of energy, the way of pre-processing, and product distribution to the point of sale. A total of 29 farms, providing 50.60 ha of farmland for local production of vegetable and fruit, were investigated through interviews and field visits (Fig. 1). The data set used for the LCA analysis, including the area and yield, the consumption of materials and energy, the way of pre-processing and transportation, as well as the carbon emission coefficients, was summarized in the inventory in Table 2, Tables 3A–3C.

### Conceptual framework

**Figure 2:**
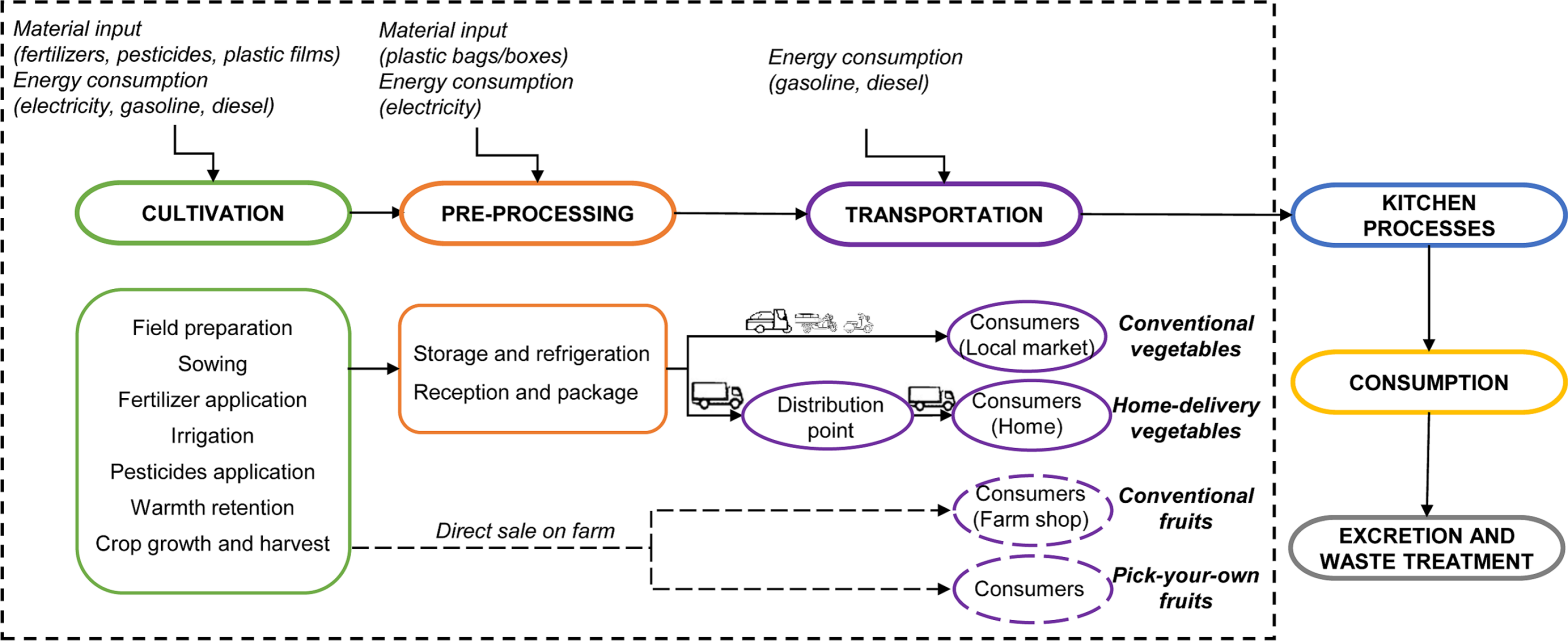
System boundaries of the urban agriculture in Beijing.

The major life-cycle stages of investigated four types of urban agriculture (Fig. 2) were summarized and reconstructed into six stages by examining the 29 real farms operating in Beijing and their production and distribution practices. The cradle to consumption stages included the cultivation, preprocessing and transportation before the vegetables and fruits were delivered to the consumers. After they were delivered to the consumers, kitchen processes, consumption and waste treatment were the main consumption to grave stages. The cultivation stage included energy costs associated with the material inputs on the farms, and the energy consumption during the on-field operations. The preprocessing stage included energy used associated with package, storage, and refrigeration. The transportation stage referred to the energy consumption during transporting the vegetables and fruits from the field to the consumer. The kitchen processes and consumption stage considered the energy consumption during the domestic cooking and eating processes. Excretion and waste treatment stages included the energy used in waste recycle and disposal.

**Table 2:** Description of four types of farms and inventory of the products (fresh vegetables/fruits).

	Farm type	Sample size	Average area (ha)	Average number of crop types	Average yield (t/ha)	Main productions	Supply
VC	Conventional smallholder operation; Greenhouse vegetable	11	0.2170	2.09	128.44	Tomato, cucumber, lettuce, cowpea, bitter gourd.	Direct sale in local markets
VN	HDA initiative; Greenhouse vegetable	6	3.9087	18.83	76.24	Cucumber, tomato, pepper, eggplant, cowpea, Chinese cabbage, zucchini, bitter gourd, etc.	Home-delivery distribution without intermediaries
FC	Conventional smallholder operation; Greenhouse fruit	7	0.1829	1.14	31.57	Strawberry, grape	Direct sale in farm shops
FN	PYO initiative; Greenhouse fruit	5	4.7467	2.20	28.54	Strawberry, grape, watermelon	Pick your own distribution without intermediaries

**Table 3:** Carbon emission coefficients, inventory of the material and energy inputs of different agriculture modes.

3A. Carbon emission coefficients of the material and energy inputs.					
Particulars	Inputs	Explanations	Unit	Carbon emission coefficients	
				kg CO_2_-eq unit^−1^	Ref.
1. On-field operations					
1) Field preparation					
	Diesel	Plowing machine	kg	3.211	NBSC (2017)
	Gasoline	Plowing machine	kg	3.243	NBSC (2017)
2) Fertilizer application					
Organic fertilizer	Manure (fresh)		t	25.667	Lal (2004)
	Manure (dry)	Dry solids	kg	0.818	Zhang et al. (2017)
Chemical fertilizer	N		kg	13.5	Zhang et al. (2013)
	P		kg	2.332	Chen, Lu & Wang (2015)
	K		kg	0.660	Chen, Lu & Wang (2015)
3) Pesticide application					
	Insecticide	Active material	kg	18.084	West & Marland (2002)
	Fungicide	Active material	kg	18.986	West & Marland (2002)
4) Irrigation					
	Electricity	Water pump	kWh	1.246	NDRC (2011)
5) Warmth retention					
	Greenhouse cover	Plastic film	kg	18.993	Tian & Zhang (2013)
	Mulching film	Plastic film	kg	18.993	Tian & Zhang (2013)
	Electricity	Shutter machine	kWh	1.246	NDRC (2011)
2. Pre-processing and transportation (VC and VN)					
1) Refrigeration and Storage	Electricity	Refrigerator, freezer	kWh	1.246	NDRC (2011)
2) Package	Plastic package	Plastic bag or box	kg	18.993	Tian & Zhang (2013)
3) Transportation	Diesel	Diesel tricycle	kg	3.211	NBSC (2017)
	Gasoline	Microvan, motorcycle, gasoline tricycle	kg	3.243	NBSC (2017)

3B. Inventory of the on farm operations of different agriculture modes.						
Particulars	Explanations	Unit Per ha	VC	VN	FC	FN
1. Material inputs						
1) Fertilizer						
Organic		t	46.31	45.50	72.74	47.22
Chemical		kg	515.26	96.19	0.00	0.00
2) Pesticide	Active material	kg	14.26	2.82	2.31	1.66
3) Plastic agricultural film	Greenhouse cover and mulching film	kg	1441.16	1236.19	1319.72	1199.25
2. Energy consumption						
1) Petroleum energy (diesel and gasoline)	Plowing machine	kg	66.50	72.30	46.14	54.97
2) Electricity	Irrigation and warmth retention	kWh	3873.74	3779.39	3661.13	3740.30

Table 3C			
Inventory of the pre-processing and supply chains of different agriculture modes.			
Particulars	Explanation and assumptions	Unit Per kg	Min.–Max. (10^−3^)
1. Pre-processing and local sale (VC)			
1) Refrigeration and Storage	Electricity used in refrigerator and freezer	kWh	2.73
2) Package	Plastic woven bag	kg	1.62
3) Transportation	Diesel tricycle	kg	27.06
2. Pre-processing and home-delivery (VN)			
1) Refrigeration and Storage	Electricity used in refrigerator and freezer	kWh	23.24
2) Package	Plastic case in bag or box	kg	3.02
3) Transportation			
Farm - Distribution point	Frequency: 52∕104∕156 times every year. Gasoline consumption (CCAG, 2016; RBJAC, 2018; SGMW, 2016): Min.:5.8 L/100 km, Max.:9.8 L/100 km. Determined by the vehicle’s fuel consumption per 100 km and the transportation distance	kg	20.38–25.30
Distribution point - Home	1 serving (5 kg). Min.: 25% by car. Max.: 75% by car. The rest: by foot	kg	1.51–5.62

### LCA, system boundaries and functional unit

A cradle to consumption LCA was conducted to estimate the carbon emissions in conventional smallholder operation and innovative largescale farming of vegetable and fruit cultivation in Beijing. The system boundaries contained two components: (1) the cradle to farm gate, including the manufacture of the agricultural material inputs (e.g., fertilizer, pesticide, plastic films, etc.) and the energy consumption during vegetable and fruit cultivation operations (e.g., sowing, irrigation, harvest, etc.) on the farm and (2) the farm gate to consumption, encompassing the material inputs and fuel consumption in the pre-processing and transportation from farms to consumers (Fig. 2). Both modes of fruit production in this study directly sold fruits right on the farm, thus the carbon emissions of pre-processing and transportation were negligible. As for the vegetable production, the carbon emissions of the plastic bags or boxes used in the package, the electricity consumed in refrigeration, and the gasoline and diesel consumed by the vehicles in the transportation were estimated.

The functional unit is the reference unit for the inventory development, carbon estimation, and comparison of the different urban agriculture modes. For a better understanding of the carbon emission results of agricultural production, a double functional unit was used in the assessment of the on-farm (cradle to farm gate) stage: the mass unit (kilograms) and the planting area (hectares) of the vegetable/fruit produced. For the post-farm (farm gate to consumption) stage, the functional unit was the mass unit (kilograms) of the vegetable/fruit consumption. The final functional unit defined for the analysis from cradle to consumption was the mass unit (kilograms) of the vegetable/fruit produced as well.

### Inventory development and assumptions

#### Primary production

The cultivation stage contained the entire processes from seeding to harvest. As all the cases sowed and harvested by hands rather than machines, the carbon emissions of sowing and harvest were ignored. The carbon emissions of respiration and the carbon absorption of photosynthesis during the crop growth were also not considered, since these would be negligible compared to the total carbon emissions of the vegetable and fruit production.

Fertilizers, pesticides, and plastic films were the major material inputs in the plastic covering greenhouse production. Data were collected as previously described in Hu et al. (2019). The amounts of nitrogen (N), phosphorus (P) and potassium (K) fertilizers (Tables 3A and 3B) were calculated by the dosage of the compound fertilizer and the percentage of each nutrient. According to a study reported on *PNAS* (Zhang et al., 2013), the carbon emission coefficient of N fertilizer production and application in China was estimated to be 13.5 t CO_2_-eq/t. The carbon emission coefficients of China’s P and K fertilizers were obtained from the estimation at the national general level (Chen, Lu & Wang, 2015). The carbon emission factor of manure dry matter was based on a study on the CF of grain production in China (Zhang et al., 2017), while the coefficient of fresh manure was obtained from the Lal’s (2004) review on the carbon emission from farm operations. Similarly, the amount of pesticide (Tables 3A and 3B) was calculated by the amount of pesticide products used and the percentage content of the active ingredients. The specific brands of the same type of pesticides were not differentiated. Given the lack of research on carbon emission from pesticide in China, the coefficients of pesticide (insecticide and fungicide) used in this work were determined by the study conducted in the USA (West & Marland, 2002), including the whole process of pesticide formulation. Both greenhouse cover and mulching film used in all cases were plastic films, and the factor was derived from the work of Tian & Zhang (2013) on the agricultural plastic film in China.

In addition to material inputs, the cultivation process also consumes energy, mainly in plowing, irrigation and warmth retention. The plowing machine consumed gasoline or diesel according to the type of equipment. Carbon emission factors of diesel and gasoline were calculated by the conversion factor to standard coal and the carbon emission coefficient of standard coal, which both were from China Energy Statistical Year Book (NBSC, 2017). Since all the cases were not equipped with any independent power supply facilities, and the electricity they used came from the unified electric power system, it was assumed that the electricity consumed in each case is generated from the same source with the same carbon emission coefficient. The coefficient used in this study was the overall carbon emission factor of power supply units in North China Regional Power Grid (including Beijing, Tianjin, Hebei, Shanxi, Shandong and west of Inner Mongolia), which was derived from the Provincial GHG Inventory Guidelines of China (NDRC, 2011). The carbon footprint calculation and the emission parameters selection for the carbon footprint calculation was clearly introduced in the supplement material (SI).

### Pre-processing and distribution

The carbon emission of package came from the use of packing materials, usually plastic bag or box, and the refrigeration and storage produced carbon emissions through electricity consumption. The carbon emission of the distribution phase mainly came from the energy consumption during the transportation from the field to the market. The embodied carbon emission from manufacture of the vehicles was beyond the scope of this analysis.

In conventional smallholder operation, the harvested vegetables are usually transported from the individual farm to the nearby markets immediately and directly sold to consumers. The vegetable distribution of HDA initiative was simplified analyzed combining the main concentration areas of customers with the number of consumers in each region, because specific home address of each customer was usually regarded as a trade secret and hard to obtain (Table 3C). Consumers of HDA initiative mostly concentrated in several different residential districts. The place where a number of consumers located was seen as a “distribution point”, and consumers live near the distribution point were seen as a “consumer group”. The vegetable distribution was organized in units of consumer groups. The deliveryman picked up all the servings of the group, took them to the distribution point by microvan or car and then delivered the servings from door to door by foot, by bicycle or, to a lesser extent, by microvan or car. It was assumed that the consumers in the same group are 1 km away from each other. After all the deliveries in one consumer group were completed, the deliveryman drove the microvan/car back to the farm and prepared the vegetables for the deliveries of next group.

As for the fruit supply, the carbon emissions of pre-processing and distribution stages were negligible since fruits of both modes were sold on farm. The conventional smallholder operations sell their fruits right in the farm shop and the fruits were just picked by consumers’ hands in the PYO initiatives. Carbon emissions from the consumers’ journey to the farm were beyond the scope of this analysis.

### Carbon emission reduction potential estimation

Using jatropha-based biodiesel in place of current petroleum, and using hydro-powered electricity instead of the current fossil-fuel-dominated electricity while other conditions were held constant, a simple hypothetical simulation was conducted to further analyze the reduction potential of carbon emission from urban agriculture in Beijing. Ou et al. (2009) assessed the GHG emissions of current six biofuel pathways in China, and found that the emission of jatropha-based biodiesel is 50.66% and 50.01% of that of conventional diesel and gasoline, respectively. According to the estimation of China’s eight electricity generation technologies (Feng et al., 2014), the total life-cycle carbon emission of electricity generated from hydropower was far lower than that of fossil-fuel based electricity, at only 13.2 g/kWh.

## Results

### CF of conventional smallholder operation and innovative largescale agriculture in Beijing

#### Vegetable production: conventional versus HDA initiative

**Figure 3:**
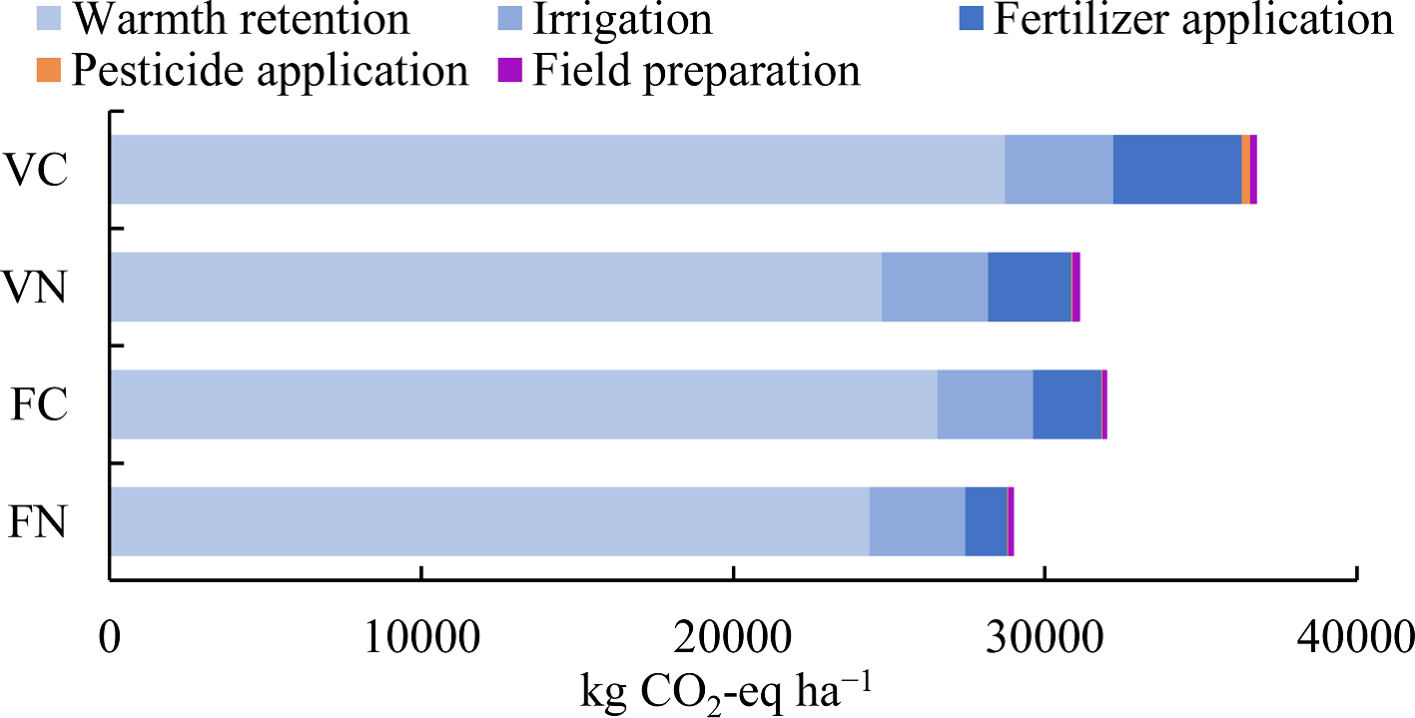
Carbon footprint of four urban agriculture modes in Beijing: from cradle to farm gate. The abscissa axis (*x*-axis) was the CF (unit: kg CO2-eq ha −1) and the length of the bar in different colors represented the CF of different sections (i.e., field preparation, fertilizer application, pesticide application, irrigation and warm retention) from cradle to farm gate in four urban agriculture modes (i.e., VC, VN, FC and FN).

The CF per area of cultivation in on-farm operation was estimated at 36,784 and 31,110 kg CO_2_-eq ha^−1^ for VC and VN, respectively (Table 4, Fig. 3). It indicated that the CF of cultivating 1 ha of vegetables in HDA initiative is about 15.4% lower than that of conventional smallholder vegetable farm in Beijing. The warm retention, including the material inputs of greenhouse plastic cover and mulching film and the electricity consumption of shutter machine, was the largest carbon emitter of the greenhouse vegetable cultivation in Beijing, accounting for 78.1% and 79.6% in VC and VN, respectively. The second hotspot of CF in VC was fertilizer application (11.2%, 4112 kg CO_2_-eq ha^−1^) followed by irrigation (9.4%, 3,475 kg CO_2_-eq ha^−1^). However, the CF of fertilizer application (8.5%, 2,637 kg CO_2_-eq ha^−1^) in VN was lower than irrigation (11.0%, 3,412 kg CO_2_-eq ha^−1^). Although fertilizer application was a major CF contributor for both VC and VN, there were some differences in the specific sources. More than 70% of the CF of fertilization in VC came from chemical fertilizer, while manure accounted for about 80% of the CF of fertilization in VN. Regarding to the pesticide application, the CF in VC was 259 kg CO_2_-eq ha^−1^, more than five times of that in VN (51 kg CO_2_-eq ha^−1^).

The CF of pre-processing and distributing vegetables in Beijing was estimated at 0.1210 and 0.1574−0.1866 kg CO_2_-eq kg^−1^ in VC and VN, respectively (Table 4). It showed that the CF per unit of yield in off-farm phase of HDA initiative is about 38.8%-45.9% higher than that of conventional smallholder operation. Transportation, followed by package, was the primary contributor of the CF from farm gate to consumption for both two modes and was estimated to be 0.0869, 0.0710−0.1002 kg CO_2_-eq kg^−1^ in VC and VN, respectively. The CF of package in VN was estimated at 0.0574 kg CO_2_-eq kg^−1^, almost twice of that in VC (0.0307 kg CO_2_-eq kg^−1^). Storage and refrigeration contributed 15.5%-18.4% (0.0290 kg CO_2_-eq kg^−1^) of the CF from farm gate to consumption in VN, while only 2.8% (0.0034 kg CO_2_-eq kg^−1^) in VC because vegetables were sent to local market by smallholders immediately after harvest and they rarely need to be refrigerated.

**Table 4:** Carbon footprint of greenhouse vegetable and fruit production systems in Beijing: from cradle to consumption.

	CF per unit of area (Unit: kg CO_2_-eq ha^−1^)				CF per unit of yield (Unit: kg CO_2_-eq kg^−1^)			
Particulars	VC	VN	FC	FN	VC	VN	FC	FN
1. On-farm phase								
1) Field preparation	214	234	148	178	0.0017	0.0031	0.0047	0.0062
2) Fertilizer application	4112	2637	2177	1355	0.0321	0.0345	0.0689	0.0475
Manure	1189	2108	2177	1355	0.0093	0.0276	0.0689	0.0475
Chemical fertilizer	2923	529	0	0	0.0228	0.0069	0	0
3) Pesticide application	259	51	43	31	0.0020	0.0007	0.0014	0.0011
4) Irrigation	3475	3412	3082	3062	0.0271	0.0448	0.0976	0.1073
5) Warmth retention	28724	24776	26544	24376	0.2236	0.3250	0.8408	0.8542
Plastic film	27372	23479	25065	22777	0.2131	0.3080	0.7939	0.7982
Shutter machine	1352	1297	1479	1599	0.0105	0.0170	0.0469	0.0560
**On-farm subtotal**	36784	31110	31994	29002	0.2865	0.4081	1.0134	1.0163
2. Post-farm phase								
1) Storage and refrigeration					0.0034	0.0290		
2) Reception and package					0.0307	0.0574		
3) Transportation					0.0869	0.0710–0.1002		
Farm gate to distribution point					–	0.0661–0.0820		
Distribution point to home					–	0.0049–0.0182		
**Off-farm subtotal**					0.1210	0.1574–0.1866		
**Total**					0.4075	0.5655–0.5947		

The cumulative CF of vegetable production from cradle to consumption was estimated to be 0.4075 kg CO_2_-eq kg^−1^ of VC and 0.5655−0.5947 kg CO_2_-eq kg^−1^ of VN (Table 4). For both modes, the on-farm cultivation (68.6%–72.2% of the total CF) was the primary source of carbon emission, followed by transportation (12.6%–21.3%) and package (7.5%–10.2%). Among the several operations of on-farm cultivation, the most relevant emitter was the warmth retention (54.6%–57.5% of the total CF) for both two modes, followed by fertilizer application in VC (7.9%) and irrigation in VN (7.5%−7.9%).

#### Fruit production: conventional versus PYO initiative

The CF per area of cultivation in on-farm operation was estimated at 31,994 and 29,002 kg CO_2_-eq ha^−1^ for FC and FN, respectively (Table 4, Fig. 3). It indicated that the CF of cultivating 1 ha of fruits in PYO initiative is about 9.4% lower than that of conventional smallholder operated fruit farm in Beijing. However, when expressed per unit of product weight, then the CF of PYO initiative (1.0163 kg CO_2_-eq kg^−1^) was 0.3% higher than that of the conventional operation (1.0134 kg CO_2_-eq kg^−1^). Similar to the vegetable production, in fruit production, the hotspot was warmth retention for both two modes, which accounted for 83.0% and 84.0% of the total CF in FC and FN, respectively. The second highest contributor for both two modes was irrigation (9.6%-10.6%), followed by fertilizer application (4.7%−6.8%). The CF of irrigation (3,082 kg CO_2_-eq ha^−1^) in FC was slightly higher than that in FN (3,062 kg CO_2_-eq ha ^−1^), and the CF of fertilizer application (2637 kg CO_2_-eq ha ^−1^) in FC was 21.1% higher than that in FN (2177 kg CO_2_-eq ha ^−1^). The remaining contributors, like field preparation and pesticide application, were less important from the perspective of CF value and proportion for both modes.

### Sensitivity analysis

The carbon emission coefficients changes could directly influence the cumulative CF results in LCA. Thus, a sensitivity analysis was conducted to evaluate the effect of carbon emission coefficients on the cumulative CF results. According to several previous practice, the variation was set to be 10% (Liang, Xu & Zhang, 2013; Ou et al., 2009). Considering the technological progress in the future, the carbon emission factors of inputs were set to decrease rather than increase (Table 5). A 10% variation in the carbon emission coefficient of plastic films brought about 5.5%−7.5% change in the cumulative CF outcomes, which was associated with the high proportion of plastic films related CF in the total CF. The sensitivities of the total CF outcomes for the variations in the carbon emission factors of the remaining inputs mostly were around 1.0%.

**Table 5:** Cumulative CF change caused by the carbon emission coefficient (*δ*_*i*_) variation of 10% (Unit: kg CO_2_-eq kg^−1^).

	Cradle to farm gate				Cradle to consumption		
					VC	VN	
	VC	VN	FC	FN		Min.	Max.
Origin value	0.2865	0.4081	1.0134	1.0163	0.4075	0.5655	0.5947
*δ* (fertilizer) (−10%)	0.2833	0.4047	1.0065	1.0116	0.4043	0.5621	0.5913
*δ* (pesticide) (−10%)	0.2863	0.4080	1.0133	1.0162	0.4073	0.5654	0.5946
*δ* (plastic films) (−10%)	0.2650	0.3772	0.9339	0.9364	0.3831	0.5290	0.5582
*δ* (electricity) (−10%)	0.2827	0.4019	0.9990	1.0000	0.4034	0.5564	0.5856
*δ* (petroleum energy) (−10%)	0.2863	0.4078	1.0129	1.0157	0.3986	0.5581	0.5844

**Table 6:** Cumulative CF reduction using alternative energy. (Unit: kg CO_2_-eq kg^−1^).

	VC	VN	FC	FN
Origin value	0.4075	0.5655–0.5947	1.0134	1.0163
Absolute CF saving by combination	0.0849	0.1269–0.1415	0.1453	0.1647
by biodiesel	0.0443	0.0370–0.0516	0.0023	0.0031
by hydro-electricity	0.0406	0.0898	0.1430	0.1616
Relative CF saving by combination (%)	20.8	22.4–23.8	14.3	16.2

### CF reduction by using alternative energy

For vegetable production, the simulation of a switch to use biodiesel in place of gasoline and diesel reduced total CF of VC and VN by 10.9% and 6.6−8.7%, respectively. Using hydro-powered electricity instead of the current fossil-fuel-dominated electricity reduced total CF by 10.0% and 15.1–15.9% for VC and VN, respectively. The combination reduced total CF by 20.8% from 0.4075 kg CO_2_-eq kg ^−1^ to 0.3226 kg CO_2_-eq kg ^−1^ for VC and 22.4–23.8% from 0.5655−0.5947 kg CO_2_-eq kg ^−1^ to 0.4386−0.4532 kg CO_2_-eq kg ^−1^ for VN (Table 6).

For fruit production, the switch to the use of biodiesel in combination with hydro-powered electricity reduced total CF by 14.3% and 16.2% for FC and FN, respectively. Compared with vegetable production, the relative simulated reduction in the CF of fruit production was lower. On the contrary, the absolute CF saving (0.1453−0.1647 kg CO_2_-eq kg^−1^) of fruit production was higher than that of vegetable production (0.0849−0.1415 kg CO_2_-eq kg^−1^) (Table 6).

## Discussion

### Carbon footprint of conventional smallholder operation and innovative largescale agriculture

For both vegetable and fruit production, the innovative largescale farms generated lower carbon emission per area of cultivation. This is consistent with a previous comparative study of large-scale and small household farming operations for grain production in China (Zhu et al., 2018). The CF per area of cultivation in on-farm operation of HDA initiative was about 15.4% lower than that of conventional smallholder vegetable farm, and the CF per area of cultivation in on-farm operation of PYO initiative was about 9.4% lower than that of conventional smallholder fruit farm. The innovative largescale farm brought CF reduction compared with conventional smallholder operation for several reasons. Much of the difference in CF was primarily due to the high input of fertilizers in conventional fruit and vegetables production systems. Most greenhouse vegetable production operated by conventional smallholders were targeted at attaining high yield to increase income and they applied large quantities of chemical fertilizer, which is easier in application and cheaper than organic manure. Driven by the same purpose of high yield, conventional smallholder fruit production applied more manure than the PYO initiative. The second difference in carbon emissions between the conventional and innovative largescale vegetable production existed in the pesticide application. In order to obtain high yield, the dosage and accompanying carbon emission of pesticide application in conventional smallholder operation was much higher. Regular pesticide, such as imidacloprid and chlorothalonil, was often used in conventional smallholder operation, while bio-pesticide with relatively lower environmental impact, like matrine, veratrine, eugenol and bacillus thuringiensis, was applied in HDA initiative to produce pollution-free, healthy and thus above-normal priced vegetables. The gap between the CF from pesticide application of conventional and innovative largescale farms was smaller in fruit cultivation than in vegetable production. This was because the conventional smallholder fruit farming with direct sale in farm shops profited from providing handy fresh, and relatively expensive fruits rather than obtaining high yields, and this commercial strategy is similar with the innovative largescale PYO initiative. Thirdly, one of the main advantages of the innovative largescale farm over the conventional smallholder systems is in different underlying socioeconomic and technical conditions. The innovative largescale farms in cities are usually managed by specialized agricultural enterprises, and supported by the work of agronomy specialists. This makes them more able to afford the adoption of improved technologies and farming practices, which can improve the input use efficiency. Meanwhile, they make profit by offering fresh, pollution-free and above-normal priced vegetables or fruits, and additional invisible services (e.g., recreational farming experience offered in PYO initiative, saving time and effort in shopping and selection provided by HDA initiative). This commercial strategy allows for more environmentally friendly decisions on some issues, such as the fertilizer varieties and dosages, rather than purely economically orientated. However, for both vegetable and fruit production, the conventional smallholder cultivation environmentally performed better than the innovative largescale farms when the CF results are expressed per product unit. The CF per unit of yield in on-farm operation of conventional smallholder vegetable farm was 0.2865 kg CO_2_-eq kg^−1^, which was about 29.8% lower than that of HDA initiative (0.4081 kg CO_2_-eq kg^−1^). The CF per product unit in on-farm operation of conventional smallholder fruit farm was slightly lower than the PYO initiative. These inverse results are attributed to the fact that the innovative largescale farms have lower crop yields on the same cultivation area as conventional. Moreover, taking the supply chain into consideration, the gap of CF from cradle to consumption between the two modes of vegetable production was wider. The primary difference of the CF in the supply chain between the two modes was in storage and refrigeration, which exhibited much higher carbon emission in HDA initiative (0.0290 kg CO_2_-eq kg^−1^) than conventional smallholder operation (0.0034 kg CO_2_-eq kg^−1^). To meet the clienteles’ diverse demands for vegetable varieties and delivery time, HDA initiative usually send the vegetables into cold storage immediately after harvest to keep in fresh. However, taking vegetables to the nearby local markets and selling them to the consumers were a part of the smallholders’ daily round during harvest seasons, thus, storage and refrigeration was only occasionally needed. In addition, the carbon emission from package of HDA initiative was also significantly higher than the conventional smallholder operation since the package of the former was more exquisite than the latter. HDA initiative usually pack each vegetable separately in a plastic box or bag for each delivery, while conventional smallholders use rough woven bags for tens of kilograms of vegetables.

Transforming to innovative large-scale agriculture from conventional smallholder operation would reduce the carbon footprint of vegetable and fruit production with the same cultivation area. However, the innovative largescale agriculture, due to its lower yields, requires significantly larger cultivation area to achieve the equal yield with conventional operation. The additional emission may offset the carbon reduction and the total carbon emission may be greater. Given the background of urban population growth and demand upgrading of urban residents, it may not possible to prevent the burgeoning innovative large-scale agriculture from gradually substituting the conventional smallholder operation, and this agriculture transformation may lead to an increase of the agricultural carbon emission. Therefore, more effective options to increase the sustainability of the innovative large-scale agriculture systems should be investigated. Meanwhile, appropriate actions should be taken to reduce the carbon emission of conventional smallholder operation, since it currently dominates the agricultural system in China.

### Hotspot of urban agriculture in Beijing

Within the boundary from cradle to consumption, the on-farm operation stage was the main source of the carbon emission of vegetable production in Beijing, and this is consistent with several previous LCA studies (Pérez-Neira & Grollmus-Venegas, 2018; Rothwell et al., 2016). For conventional smallholder vegetable operation, the on-farm cultivation phase contributed to 70.3% of the total carbon emission, and the ratio was 68.6%-72.2% in the innovative largescale HDA. The CF from on-farm operation phase of both modes in this study(0.2865, 0.4081 kg CO_2_-eq kg^−1^) was higher than the national average carbon emission (0.06−0.21 kg CO_2_-eq kg^−1^) of vegetable production (Yue et al., 2017) since cases in this study generally adopted the high-input greenhouse production mode. Among the several operations of on-farm cultivation phase, warmth retention, fertilizer application and irrigation were the most relevant emitters. This was reflected in both conventional smallholder operation and innovative largescale farm of vegetables and fruit production. The plastic films contributed the lion’s share of the CF from cradle to farm gate of vegetable and fruit production in urban agriculture in Beijing. Bojacá, Wyckhuys & Schrevens (2014) also recognized that the polyethylene cover is the primary contributor to GWP of Colombian greenhouse tomato production with a share of 45%. Another Italian case study assessed environmental performances of five protected crops and indentified that the inputs of plastic sheet and fertilizer are key factors reveal the quantitative GWP difference between different crops (Cellura, Longo & Mistretta, 2012). The infrastructure in the above cited LCA studies was the same as that in this work, which is the greenhouse with no heating systems. The heated greenhouse production, featuring with intensive energy consumption, embodied significantly higher carbon emission and the auxiliary heating system is generally regarded as the hotspot (Almeida et al., 2014; Dias et al., 2017; Page, Ridoutt & Bellotti, 2012).

As for the off-farm stage, transportation was the major carbon emitter from farm gate to consumption of vegetable production in Beijing. It was also the second largest contributor to the total CF of both two modes, right behind warmth retention. For the conventional smallholder vegetable farm, the transportation contributed to 21.3% (0.0869 kg CO_2_-eq kg^−1^) of the total carbon emission, and the proportion was 12.6%-16.8% (0.0710−0.1002 kg CO_2_-eq kg^−1^) in the innovative largescale HDA. All the cases in this work adopted unmediated distributions and the vegetables were transported over short distances. Direct sales from farm to fork are supposed to be beneficial for the carbon emission reduction of agricultural products (Benis & Ferrão, 2017; Stoessel et al., 2012) by eliminating the energy costs associated with intermediaries (Pérez-Neira & Grollmus-Venegas, 2018). Therefore, CF from transportation in this work was quite lower than those long distance traveled vegetables. For instance, the carbon emission in transportation of fresh tomatoes traveled from Queensland to Sydney market was estimated at 0.36 kg CO_2_-eq kg^−1^, which was about three- to five-fold of this work. In addition, another emitter in supply chain which is frequently mentioned in previous works, packaging embodied quite different amount of carbon emissions between the conventional smallholder operation and the HDA initiative. Nonetheless, comparing with the tin plate cans of tomato products (0.447 kg CO_2_-eq kg^−1^) (Theurl et al., 2014) and other multiple packaging (0.491−0.826 kg CO_2_-eq kg ^−1^) (Del Borghi et al., 2014), the CF from packaging in both modes is relatively lower because the packaging of fresh vegetables is more simplified than that of processed products in those mentioned studies.

### Policy implications

As a permanent and dynamic part of the urban socio-economic and ecological system, agriculture in cities plays an important role in achieving sustainable urban development (Van Veenhuizen & Danso, 2007). Conventional smallholder-operated farms and innovative largescale agriculture systems are different forms of urban agriculture. The innovative largescale agriculture, like HDA and PYO initiatives, has been gaining popularity in metropolis in China, mostly due to the social benefits, such as recreation of agricultural tourist experience. The result of this study showed that innovative largescale agriculture could also have a better performance in reducing the CF per cultivated area than conventional smallholder operations. These innovative largescale urban agriculture systems are therefore worth embedding into the process of city planning and designing, taking into account the aesthetic values of surrounding urban landscape and the needs of local citizens for other uses of public space (Kulak, Graves & Chatterton, 2013). Moreover, the result of this research also declared that transportation is one of primary CF hotspots, thus the layout of these innovative largescale agriculture should also consider the traffic network and residential distribution to improve the transportation efficiency and thereby reduce the CF from transportation.

Many cities in China have formulated a series of policies and action programs to enhance the potential of urban agriculture and reduce the associated environmental risks. Owing to its capital status and high level of urbanization, Beijing is a pioneer that has integrated its urban agriculture developments into city development plans (Yang et al., 2016) with an emphasis on promoting the large-scale operation (BJMG, 2013; BMCRA, 2016). The CFs per product unit of the home-delivery, pick-your-own and other forms of innovative large-scale urban agriculture tend to be higher than the conventional due to the lower yields. Those innovative large-scale urban agriculture should not be seen as an ultimate solution to reduce the carbon emission associated with the food supply in current populous China and targeted actions should be taken to reduce the CF for both conventional smallholder operation and innovate large-scale farming.

Government should provide training, technical assistances and extension services to urban farmers, with an emphasis on low-carbon farming practices. Lifespan extension and recycling of the plastic materials with high CF, like greenhouse cover and mulching film, can be very beneficial to reduce the emission in on-farm stage. Reducing consumption rate and enhancing use efficiency of both chemical and organic fertilizer through scientific application are also essential. It is particularly noteworthy for small householders to use pesticides properly. As for the carbon reduction from off-farm phase, over-packaging should not be advocated since the use of materials for packaging is one of the most significant emitters in supply chain. The eco-design of packaging should be investigated and checked. One of the improvement solutions could be the use of renewable material in packaging. For instance, the current common plastic boxes and bags used for vegetables can be replaced with biodegradable plant-based plastics. These alternatives could be implemented by urban farmers without deep behavioral change of consumers.

Concerning greenhouse production, greenhouse with good performance of thermal insulation and irrigation systems is very favorable for energy saving and carbon emission reduction. Therefore, special construction projects should be established to improve the basic infrastructure and agricultural facilities. Economic and financial measures such as subsidies, tax cuts and special credit schemes can be adopted for urban farmers to promote the application of energy-efficient and low-carbon equipment and advanced resource-saving agricultural technologies. Meanwhile, renewable energy, like biofuel, solar and wind power, should be appropriately promoted in urban agriculture to reduce the energy related carbon emission. In addition, some general actions, such as encouraging further technological breakthroughs in renewable energy, would also contribute to the carbon reduction of urban agriculture.

### Research limitation and project into the future

Whilst the scope and framework (Fig. 2) developed in this study provide a cautious and satisfactory carbon emission estimation of the innovative large-scale agriculture and conventional smallholder operation using LCA, it did not fully incorporate the whole life cycle of the products. The up-stream manufacture process of infrastructures, equipment and vehicles, and the uncertain dispose of the residue and other materials (e.g., some residue were fed to chickens, some used plastic films were taken away by waste collectors) were not taken into account. The influence of driving behaviors and traffic conditions, and consumption expenses within the consumers’ home were not considered either. As a an internationally recognized tool, LCA has a specific definition (system boundaries, function units, inventory categories, etc.) in every practice and its calculation may vary from study to study. Therefore, on the premise of fully considering the methodological definition and limitation, the comparative results of this study can be interpreted and applied with analytical caution.

Although all the sample farms we chose were qualitatively typical of the typology they belong to, the numerical results cannot extrapolate to the whole city due to the limited sample size. In further analysis at regional scale, more sample cases should be investigated. Thus, whilst this paper has shown that the carbon emission per unit of cultivation area can be reduced while the carbon emissions per unit of product will increase by innovative large-scale agriculture, a regional analysis of the carbon emission change is needed to explore to what extent replacing conventional smallholder operation with those innovative large-scale farming would be beneficial. Meanwhile, many other innovative urban agriculture forms in addition to the HDA and PYO initiatives have burgeoned in Beijing, such as vegetable basket project, agricultural sightseeing gardens, community-supported agriculture, etc. Some farms integrate different commercial patterns and therefore the field management and inputs are more complex. Therefore, it is also necessary to carry out environmental impact assessment for more diverse types of urban agriculture in future studies.

This research focused on the common vegetable and fruit production systems of urban agriculture in Beijing. However, there are some advanced agricultural facilities and techniques that may be applied in innovative urban farms and that could be researched to determine their potential impacts on carbon emissions. Examples include solar photovoltaic powered pumping and lighting system, crop-animal combined production, integration of automatic irrigation and fertilization system (fertigation), soil-free growing techniques (aeroponics and hydroponics), which can potentially reduce carbon emissions. An LCA of the resulting agricultural products is required to evaluate the contribution of these techniques on carbon reduction.

## Conclusions

This analysis of greenhouse vegetable and fruit production in Beijing has shown that the innovative large-scale agriculture (home-delivery vegetable and pick-your-own fruit initiative) can produce a considerable reduction in carbon emission per area of cultivation compared with the conventional smallholder operation, by reducing the consumption of agricultural inputs (e.g., fertilizer, pesticides). In this sense, this form of innovative largescale urban agriculture is worth embedding in urban landscapes planning. However, the innovative large-scale agriculture has a higher carbon emission per product weight unit than the conventional smallholder operation due to its lower yields. Thus, the innovative large-scale urban agriculture cannot be seen as an ultimate solution to reduce the carbon emission of food supply for current China with the demand for food security. Appropriate measures on carbon reductions should be adopted for both conventional smallholder operation and innovate large-scale farming. Quantitative evaluations showed that the use of plastic materials, fossil energy dependence, and transportation efficiency are critical aspects to reduce the carbon emissions for both conventional smallholder operation and innovative large-scale farming. More attention should be paid in effective pesticide application for conventional smallholder, while the packaging may be an important target of technological advance and social habit progress in innovative large-scale home-delivery farming.

In summary, the present study provides novel information at the local level (Beijing, China) on the carbon footprint of greenhouse vegetable and fruit production. Hotspot identification was provided to recognize the particular inputs and activities which should be primarily targeted for carbon reduction adjustment of conventional smallholder operation and innovative largescale agriculture. In the course of this study, opportunities for further analysis and crucial research gaps have also been identified. Such research will further advance understanding of how innovative largescale agriculture and conventional smallholder operation compare and which primary aspects of urban agriculture should be focused on for contributing the greatest savings in carbon emissions.

##  Supplemental Information

10.7717/peerj.11632/supp-1 Supplemental Information 1 The material input and energy consumption of the four different urban agriculture types The number in the table was the average value of total consumption divided by the total cultivation area (or output) of each type.

10.7717/peerj.11632/supp-2 Supplemental Information 2 The raw data of material input and energy consumption of each investigated sample farm for each type (VC, VN, FC, and FN)

10.7717/peerj.11632/supp-3 Supplemental Information 3 The English translation of the questionnaire on arable land use and carbon effect of innovative largescale farming in Beijing (the original was in Chinese)

10.7717/peerj.11632/supp-4 Supplemental Information 4 The English translation of the questionnaire on arable land use and carbon effect of conventional smallholder in Beijing (the original was in Chinese)

10.7717/peerj.11632/supp-5 Supplemental Information 5 Appendices Carbon footprint calculation of the four urban agriculture modes and Factor selection for the carbon footprint calculation.

## Additional Information and Declarations

### Data Availability

The following information was supplied regarding data availability: The raw measurements are available in the Supplemental File.
