# Supplementary material for: Comparative analysis of carbon footprint between conventional smallholder operation and innovative largescale farming of urban agriculture in Beijing, China
Source: PeerJ. 2021 Jun 29;9:e11632. doi: 10.7717/peerj.11632 (PMC8253110; doi:10.7717/peerj.11632)
Supplement: Supplemental Information 3 [file peerj-09-11632-s003.pdf]

北京市规模经营农业园区土地利用及碳足迹调查问卷（问卷编号：__________）

_______区_______镇_______村；日期________；调研员_______；北纬___________东经_________高程________；

尊敬的受访者：

您好，因北京市自然科学基金重点项目（项目名称：基于低碳北京的土地利用变化碳效应过程模拟及空间优化配置研究，项目编号：8151001）研究需要，现通过问卷调查的形式向您咨询耕地利用及碳效应相关信息。问卷中所有信息仅供研究使用。所有的数据均是匿名处理和分析的，问卷所涉及的个人信息仅为了解园区基本情况所用，在未经过您允许的前提下，不会被泄露至第三方。

北京市自然科学基金重点项目研究小组

Questionnaire on Arable Land Use and Carbon Effect of Innovative Largescale Farming in Beijing (No: ________)

District: _________ Town: _________ Village: _________; Date: _________; Interviewer: _________;
Latitude: _________ Longitude.: _________ Altitude.: _________

Dear Interviewees:

We consult you with the related information of arable land use through questionnaires for the research needs of the Key Project of Beijing Municipal Natural Science Foundation “Spatial optimizing land use allocation based on the simulation on carbon effects of land use change for low carbon Beijing” (No. 8151001). All the information in the questionnaire is only for research purposes and the data are processed and analyzed anonymously. The personal information involved in the questionnaire is for the purpose of understanding the basic situation of farming park and will not be disclosed to third parties without your permission.

Beijing Natural Science Foundation Key Project Research Group.

1. 基本情况 Basic features

（1） 园区名称 agriculture park name____________，园区类型 agriculture park type___________（A：工厂化作物栽培 factory crop cultivation，B：规模化种植业 large-scale planting，C：规模化畜禽养殖业: large-scale livestock and poultry breeding，D：现代多功能农业 modern multi-functional agriculture，E：其他 others ____________）；（可做补充介绍 Supplementary introduction____）

（2） 建设时间 build time ____；固定投资总额 total fixed investment：__万元；年生产总值 gross annual production：___万元；总面积 total area ____亩；常驻员工数量 number of permanent staff ____；临时工作人员数量 number of temporary staff ___

（3） 您在园区的职位是 your position in the park_________，您的联系方式是 your contact information：_____________

2. 土地利用情况（图上标明功能区）Current status of land use (mark the functional areas on the map)

土地利用类型 land use type	面积（亩） area	取得方式（租金） access(rent)	备注 comment	土地利用类型 land use type	面积（亩） area	取得方式（租金） access(rent)	备注 comment
耕地-大棚 farmland with greenhouse			**注明大棚类型** label the type of greenhouse	草地 grassland			**注明草地类型** label the type of grassland
耕地-无大棚 farmland -no greenhouse				建设用地 construction land			**注明容积率和用途** label the area for different uses
园地 orchard				水域 water			
林地 forest			**注明林地类型** label the type of forest	其他用地 other land			

3. 碳足迹 Carbon footprint (pre-farm，on-farm，post-farm)

3.1 能源、物质输入 energy and material inputs (pre-farm)

能源/物质投入类别 type	用量及价格 consumption and expense	备注 comment
水	总量 total consumption：________吨 t	

Water	口自来水 tap water /____________吨 t ___________元/吨 yuan/t<br>口井水 well water /____________吨 t ___________元/吨 yuan/t<br>口其他 other /____________吨 t ___________元/吨 yuan/t	
电力<br>Electricity	总量 total consumption：_________度 kwh<br>口统一供电 municipal unified power supply /______度 kwh ______元/度 yuan/kwh<br>口太阳能 solar power /______度 kwh ______元/度 yuan/kwh<br>口其他 other /______度 kwh ______元/度 yuan/kwh	
燃气<br>Gas	口管道天然气 pipeline natural gas /______方 cubic meter _____元/方 yuan/cubic meter<br>口罐装气 filling liquefied gas /______斤 jin= half kg _____元/斤 yuan/jin<br>口沼气 marsh gas /______方 cubic meter _____元/方 yuan/cubic meter<br>口其他 other /______方 cubic meter _____元/方 yuan/cubic meter	
肥料<br>Fertilizer	口外购有机肥 bought organic manure /______千克 kg _______元/千克 yuan/kg<br>口自行堆肥 homemade organic manure /______千克 kg _______元/千克 yuan/kg<br>口化肥 chemical fertilizer<br>口复合肥 compound fertilizer /_______千克 kg _______元/千克 yuan/kg<br>口尿素 urea /_______千克 kg _______元/千克 yuan/kg<br>口二胺 diamine /_______千克 kg _______元/千克 yuan/kg<br>口其他 other _________/_______千克 kg _______元/千克 yuan/kg<br>口其他 other____________	**注明有效成分含量；有机肥注明具体种类：鸡鸭猪牛**<br>The nutrient content ratio of chemical fertilizer should be indicated. Indicate specific type of organic manure: chicken/duck/pig/cow
农药<br>Pesticide	口杀虫剂 insecticide：名称 name1____；用量 dosage 1___毫升 ml；___元/毫升 yuan/kg<br>名称 name2____；用量 dosage 2___毫升 ml；___元/毫升 yuan/kg<br>口除草剂 herbicide：名称 name1____；用量 dosage 1___毫升 ml；___元/毫升 yuan/kg<br>名称 name2____；用量 dosage 2___毫升 ml；___元/毫升 yuan/kg<br>口其 他 other： 名称 name1____；用量 dosage 1___毫升 ml；___元/毫升 yuan/kg<br>名称 name2____；用量 dosage 2___毫升 ml；___元/毫升 yuan/kg	**注明有效成分含量**<br>The proportion of active ingredients of each pesticide should be noted
饲料<br>feed	口配合料 formula feed： 名称 name____；用量 dosage____吨 t；_____元/吨 yuan/t<br>口浓缩料 concentrated feed：名称 name____；用量 dosage____吨 t；_____元/吨 yuan/t<br>口预混料 premixed feed： 名称 name____；用量 dosage____吨 t；_____元/吨 yuan/t<br>口麸皮 bran： 用量 dosage____吨 t；_____元/吨 yuan/t<br>口玉米 corn： 用量 dosage____吨 t；_____元/吨 yuan/t<br>口其他 other： 名称 name____；用量 dosage____吨 t；_____元/吨 yuan/t	
其他<br>other	口大棚膜 greenhouse cover： 用量 dosage_____千克 kg；______元/千克 yuan/kg<br>口地膜 mulching film： 用量 dosage_____千克 kg；______元/千克 yuan/kg<br>口其他 other：名称 name______；用量 dosage_____千克 kg；______元/千克 yuan/kg	

3.2 生产、服务活动 production and service activities（on-farm）

3.2.1 产品部分 products

1）种植业 planting

农产品类型<br>type	名称<br>name	年播种面积<br>sown area	年产量<br>yield	粪肥施用量（kg/亩）<br>manure application	化肥施用（kg/亩）<br>chemical fertilizer application	燃油消耗（L/亩）<br>fuel consumption	农药使用量<br>pesticide application	人工（人·日）<br>labor input	秸秆处理方式<br>residue treatment/	生产过程减排措施<br>reduction measure
减排措施包括：1、滴灌；2、水肥一体化系统；3、机械尾气净化；4、其他__________ Emission reduction measures in production include: 1- drip irrigation; 2. integrated system of water and fertilizer; 3, mechanical tail gas purification; 4- others_______										
粮食<br>grain	小麦<br>maize									
	玉米<br>wheat									
蔬菜及食										

用菌 vegetable/ edible mushrooms										
瓜果 melons and fruits										
园林/花卉 nursery/flo wer garden										
其他 other										

2）养殖业 breeding

动物种类 type	存栏量 stock	饲养方式 **feeding way** **1-散养** free range **2-圈养** in captivity	饲料用量 feed consumption	粪便量及处理方式 waste amount and treatment	备注-减排措施 comment- reduction measure
牛 cow					
羊 sheep					
猪 pig					
鸡 chicken					
鸭 duck					
其他 other					
**注：粪便处理方式包括 1-沼气；2-堆肥；3-出售；4-其他** note. waste treatments inchlude1-making biogas;2-composting;3-sell;4- other					

3.2.2 服务部分 service

服务类型 type	年服务人次 annual reception	人均消费 consumption per person	碳排放源 carbon source	备注-减排措施 comment- reduction measure
住宿 accommodation				
餐饮 catering				
采摘 pick-your-own				
教育、观光 education, sightseeing				
其他 other				

3.3 产品销售 sale of products (post-farm)

产品类型 type	名称 name	年销售量 annual sale quantity	年销售额（万元） annual sale (10000 yuan)	销售目的地 1-销售量-运输方式-距离-耗油量 sale destination 1- amount – delivery means- distance- fuel consumption	销售目的地 2-销售量-运输方式-距离-耗油量 sale destination 2- amount – delivery means- distance- fuel consumption	备注 comment
粮食 grain	小麦 maize					
	玉米 wheat					
蔬菜及食用菌 vegetable/						

edible mushrooms						
瓜果 melons and fruits						
园林/花卉 nursery/flower garden						
牛 cow						
羊 sheep						
猪 pig						
鸡 chicken						
鸭 duck						

4. 对低碳土地利用的认识 Awareness of low-carbon land use

（1） 您是否听说过全球变暖？_______是否有此感受？_______1-是；2-否
Have you heard about global warming? _______ Do you have this feeling? _______1 - yes;2 - no

（2） 据您了解，引起全球变暖的原因是什么？According to your knowledge, what is the cause of global warming__________(可多选 Multi-choice)
1-化石燃料燃烧 fossil fuel combustion；2-森林砍伐 deforestation；3-人口增加 population growth；4-气候自然变化 natural climate change；5-秸秆燃烧 straw burning；6-过度施肥 over fertilization；7-其他 others________

（3） 您认为自身与节能减排有关系吗？Do you think you are responsible to energy conservation and emission reduction_______1-是 yes；2-否 no；3-不了解 not sure；
如有，您认为主要体现在哪些方面？If yes, which aspects do you think are mainly reflected in ____________1-化肥施用 fertilizer application；2-农机使用 machinery use；3-车辆排放 vehicle emissions；4-垃圾处理 waste disposal；5-教育宣传 e education and propaganda；6-其他 other________
关于节能减排，您目前有无享受相关鼓励政策？______1-有；2-无。Regarding energy conservation and emission reduction, have you ever enjoyed relevant encouragement policies so far? 1- yes;2 -no.
如有，是何政策？ If yes, what’s that. ____

（4） 关于节能减排，您有何建议？Do you have any comments and suggestions on energy saving and emission reduction?
____
____
