# Supplementary material for: Comparative analysis of carbon footprint between conventional smallholder operation and innovative largescale farming of urban agriculture in Beijing, China
Source: PeerJ. 2021 Jun 29;9:e11632. doi: 10.7717/peerj.11632 (PMC8253110; doi:10.7717/peerj.11632)
Supplement: Supplemental Information 4 [file peerj-09-11632-s004.pdf]

北京市传统小农经营耕地利用及碳效应调查问卷（问卷编号：___________）

_______区_______镇_______村；日期________；调研员_______；北纬___________东经_________高程________；

尊敬的受访者：

您好，因北京市自然科学基金重点项目（项目名称：基于低碳北京的土地利用变化碳效应过程模拟及空间优化配置研究，项目编号：8151001）研究需要，现通过问卷调查的形式向您咨询耕地利用及碳效应相关信息。问卷中所有信息仅供研究使用。所有的数据均是匿名处理和分析的，问卷所涉及的个人信息仅为了解农户基本情况所用，在未经过您允许的前提下，不会被泄露至第三方。

北京市自然科学基金重点项目研究小组

Questionnaire on Arable Land Use and Carbon Effect of Conventional Smallholder in Beijing (No: ________)

District: ____________ Town: ___________ Village: ___________; Date: ___________; Interviewer: ___________;
Latitude: ___________ Longitude.: ___________ Altitude.: ___________

Dear Interviewees:

We consult you with the related information of arable land use through questionnaires for the research needs of the Key Project of Beijing Municipal Natural Science Foundation "Spatial optimizing land use allocation based on the simulation on carbon effects of land use change for low carbon Beijing" (No. 8151001). All the information in the questionnaire only is for research purposes and the data are processed and analyzed anonymously. The personal information involved in the questionnaire is for the purpose of understanding the basic situation of farmers and will not be disclosed to third parties without your permission.

Beijing Natural Science Foundation Key Project Research Group.

一、 农户基本特征 Basic features

（1）您的年龄_____岁，性别_____，受教育水平____

Age _____, Gender _____, Education ____

（1-小学以下 below primary school；2-小学 primary school；3-初中 middle school；4-中专 technical secondary school；5-高中 high school；6-专科 junior college；7-本科 bachelor degree；8-本科以上 master degree or above）

（2）您家户籍人口______人，常住人口_____人，劳动力______人，务农____人，兼业____人，非农____人。

The registered population of your family_______, permanent residents_______, labor force________, farm labor_______, part-time labor force_______, off-farm labor_______

（3）家庭年总收入（毛收入）______元，其中农业收入______元；总支出______元，其中农业支出______元。

The annual total household income (gross income) ________yuan, of which the agricultural income ______yuan; Total expenses______yuan, including agricultural expenses______yuan.

（4）您在北京从事农业生产已有_____年，户籍所在地是_______省_______市，您的联系方式是：__________________

You engaged in agricultural production in Beijing for _____ years, your domicile place is ________ province _________city, your contact information is: _____________

二、耕地利用现状 Current status of cultivated land use

（1）您家在种耕地_____亩，其中自有____亩，租种____亩；粮食作物____亩，蔬菜作物____亩，瓜果作物____亩，其他作物____亩。

The area of land under cultivation of your household is ________, including owned_______ and rented________. The area of grain crops is______, vegetables ______, fruits______, and other crops______.

作物类型（请√）<br>CROP TYPE (√)	粮食作物<br>grain crops		蔬菜/食用菌<br>vegetable/ edible mushrooms			水果<br>fruits			其他<br>others
作物名称<br>CROP NAME	玉米<br>maize	小麦<br>wheat							
播种面积（亩）sown area (mu)									

总产量（斤）total output (jin=half kg)										
出售价格（元/斤）sale price(yuan/jin)										
种子/种苗<br>seed/seedling	费用（元）cost(yuan)									
	用量（斤）dosage(jin)									
化肥施用<br>chemical fertilizer application	费用（元）cost(yuan)									
	尿素（斤）urea(jin)									
	碳铵（斤）ammonium bicarbonate (jin)									
	二胺（斤）diamine (jin)									
	复合肥(斤)compound fertilizer (jin)									
	总量（斤）total dosage (jin)									
	**注：如化肥种类未在上表中列出，请按实际情况写明种类和用量，复合肥需写明各养分含量占比。**<br>note: if the type of chemical fertilizer used is not listed above, please fill in the type and dosage truly and correctly. The ratio of nutrient content of compound fertilizer should be indicated.									
农家肥/有机肥<br>manure/ organic fertilizer	鸡、猪、牛或其他（方、斤）chicken/pig/cow manure, or other organic fertilizer (cubic meter, jin)									
	价格（元/方、元/斤）price(yuan/jin, yuan/ cubic meter)									
	占施肥比例(percentage in the total fertilization)									
农药<br>pesticide	费用（元）cost (yuan)									
	用量 1（ml）、农药名 1<br>Dosage1(ml), pesticide name 1									
	用量 2（ml）、农药名 2<br>Dosage2(ml), pesticide name 2									
	用量 3（ml）、农药名 3<br>Dosage3(ml), pesticide name 3									
	**注：各农药需写明有效成分含量占比。**note: The proportion of active ingredients of each pesticide should be noted									
机械<br>machinery	费用（元）cost(yuan)									
	柴油 1（斤、升）、环节 1<br>Diesel 1(jin, L), process1									
	柴油 2（斤、升）、环节 2<br>Diesel 2(jin, L), process2									
	柴油 3（斤、升）、环节 3<br>Diesel 3(jin, L), process3									
	旋地深度（cm）<br>ploughing depth (cm)									
	**注：1.重点为油耗投入，若无直接数据，通过马力（瓦特）和用时（小时/亩）折算；2.环节包括灌溉、旋地、收获等**<br>note: 1. The focus is fuel consumption input. If the direct data is not accessible, it can be converted from horsepower (watt) and working time (hour/mu);2. The processes include irrigation, land rotation, harvesting, etc.									
农作物残体处理<br>residue treatment	费用 cost(yuan)									
	方式 treatment									
	**注：处理方式包括 1.秸秆还田；2.堆肥；3.就地堆放；4.其他。费用中需注意标注清楚，以免重复计算等。**<br>note: the residue treatments include 1-straw returning, 2-composting, 3-stacking on site and 4-other. Expenses should be noted clearly to avoid double counting.									
灌溉<br>irrigation	费用（元）cost(yuan)									
	用电量（度）electricity consumption (kwh)									
	用油量（斤、升）fuel consumption (jin, L)									
	**注：用电量和用油量均为总量，可通过单次消耗乘以次数计算得出；灌溉可用电或用油，注意用油与上行机械区分；**									

	note: both the electricity consumption and fuel consumption are total, which can be calculated by multiplying the number of irrigation times by a single consumption. Irrigation may consume electricity or petroleum, pay attention to the difference between the fuel consumption here and that in previous machinery. Avoid double counting or omission.									
劳动力投入 Labor input	工作日（天•人） working days (day·person)									
总投入（元）total input(yuan)										
净收益（元）net income(yuan)										

（2）您家农产品主要销往_______**（1-附近农贸市场；2-有人上门收；3-采摘；4-就地售卖；5-其他）**

如选 1，您去附近农贸市场所使用的交通工具是____**（1-人力三轮车；2-摩托车；3-面包车；4-大卡车）**，运输距离约为____，耗油量约为____

Your farm produce is mainly sold to _______ (1- nearby market;2- someone came to collect;3 – pick-your-own;4- selling on site;5-other). For choice 1, the vehicle you use to get to the nearby market is ____ (1- man-powered tricycle;2- motorcycle;3- micro van;4- large truck) with a transportation distance of about ______ and fuel consumption of about _______

三、农户非农业生产的能源消费情况 Household energy consumption in non-agricultural production

能源 energy		现状 status	减排措施 Reduction measure	能源 energy		现状 status	减排措施 Reduction measure
生活用电 domestic electricity	消费量(度/年) consumption (kwh/yr)			汽车燃油 Vehicle fuel	里程(公里/年) mileage (km/yr)		
	总费用/元 expense/yuan				单位公里油耗 Fuel consumption per kilometer		
煤炭 coal	消费量(吨/年) consumption (t/yr)				总费用/元 expense/yuan		
	总费用/元 expense/yuan			其他 others	消费量（ /年） consumption (/yr)		
燃气 gas	消费量(方/年)				总费用/元 expense/yuan		
	总费用/元 expense/yuan			备注说明 note：			

减排措施包括：1-节能炕；2-无烟煤补贴；3-燃气补贴；4-其他________Emission reduction measures include: 1- energy saving kang①;2- anthracite coal subsidy;3- gas subsidy;4- others ________ ① kang is a heatable brick bed, mainly in North China

四、对低碳土地利用的认识 Awareness of low-carbon land use

（1） 您是否听说过全球变暖？_______是否有此感受？_______1-是；2-否

Have you heard about global warming?_______ Do you have this feeling?_______1 - yes;2 - no

（2） 据您了解，引起全球变暖的原因是什么？According to your knowledge, what is the cause of global warming__________(可多选 Multi-choice)

1-化石燃料燃烧 fossil fuel combustion；2-森林砍伐 deforestation；3-人口增加 population growth；4-气候自然变化 natural climate change；5-秸秆燃烧 straw burning；6-过度施肥 over fertilization；7-其他 others________

（3） 您认为自身与节能减排有关系吗？Do you think you are responsible to energy conservation and emission reduction_______1-是 yes；2-否 no；3-不了解 not sure；

如有，您认为主要体现在哪些方面？If yes, which aspects do you think are mainly reflected in ____________1-化肥施用 fertilizer application；2-农机使用 machinery use；3-家庭取暖 home heating；4-车辆排放 vehicle emissions；5-垃圾处理 waste disposal；6-其他 other________

（4） 关于节能减排，您有何意见及建议？Do you have any comments and suggestions on energy saving and emission reduction.

____

____

____
