# Supplementary material for: Comparative analysis of carbon footprint between conventional smallholder operation and innovative largescale farming of urban agriculture in Beijing, China
Source: PeerJ. 2021 Jun 29;9:e11632. doi: 10.7717/peerj.11632 (PMC8253110; doi:10.7717/peerj.11632)
Supplement: Supplemental Information 5 — Carbon footprint calculation of the four urban agriculture modes and Factor selection for the carbon footprint calculation. [file peerj-09-11632-s005.docx]

**Comparative analysis of carbon footprint between conventional smallholder operation and innovative largescale agriculture. A case study of vegetable and fruit production in Beijing, China.**

Yingjie Hu^1^, Jin Sun^2^, Ji Zheng ^3^

^1^ College of City Construction, Jiangxi Normal University, Nanchang, China

^2^ College of Surveying and Geo-informatics, North China University of Water Resources and Electric Power, Zhengzhou, China

^3^ Department of Urban Planning and Design, The University of Hong Kong, Pokfulam, Hong Kong SAR, China

Corresponding Author:

Ji Zheng ^3^

The University of Hong Kong, 10 Sassoon Road, Pokfulam, Hong Kong SAR, China.

Email address: zhengji@hku.hk

**Supplementary information**

**Appendix A *Carbon footprint calculation of the four urban agriculture modes***

**Appendix B *Factor selection for the carbon footprint calculation***

**Appendix A *Carbon footprint calculation of the four urban agriculture modes***

1. Cumulative carbon footprint (CCF) formula of each investigated farm

**CCF=**$\sum_{i} {Input}_{i}\times\delta_{i}$ (1)

Where CCF (kg CO_2_-eq per f.u.) denotes the cumulative carbon footprint from cradle to consumption; *Input_i_* (unit per f.u.) is the consumption of the *i* th input (fertilizer, pesticide, electricity, diesel, etc.); f.u. =functional unit (ha or kg); *δ_i_* (kg CO_2_-eq per unit) is the carbon emission coefficient of the *i* th input.

1. Carbon footprint calculation of different urban agriculture modes

**CF_type_=** $\sum_{\boldsymbol{j}} {CCF}_{\boldsymbol{j}}\boldsymbol{/}\sum_{\boldsymbol{j}} {f.u.}_{\boldsymbol{j}}$ (2)

Where CF_type_ (kg CO_2_-eq per f.u.) is the average carbon footprint of the specific urban agriculture mode (VC, VN, FC, FN); CCF_j_ is the carbon emission of the *j* th investigated farm belongs to the corresponding urban agriculture mode; f.u. is the cultivation area (ha) or yield mass (kg) of the *j* th investigated farm belongs to the corresponding urban agriculture mode.

**Appendix B *Factor selection for the carbon footprint calculation***

The carbon emission coefficients selected should be accurate and practical since they directly influence the carbon footprint results obtained, but they always vary from region to region, even from case to case. China-specific or Beijing-specific parameters were preferred.

**B1 *Carbon emission factors of material inputs***

The carbon emission factor for nitrogen (N), phosphorus (P) and potassium (K) fertilizers application was derived from the study at the national general level. Zhang et al. (2013) quantitatively evaluate the carbon emission for China’s N fertilizer chain through a life cycle analysis including the fossil fuel mining, ammonia synthesis, N fertilizer manufacture and post application. The result showed that the CF of China’s N fertilizer production and application was 13.5 t CO_2_-eq/t, which had been published this factor on PNAS. Similarly, Chen et al. (2015) quantified the carbon emission factors of China’s P and K fertilizers, at the national general level as well.

The carbon emission factor of manure dry matter was based on a CF study of grain production in China (Zhang et al. 2017). Based on the carbon emission for sludge composting and dung composting (fossil carbon emissions from transport, machinery operation, processing and drying and GHG emissions from composting), and the nitrogen content values of 2.18% and 1.36%, the average carbon emission factor of manure (2% for the total nitrogen content; C:N = 13:1) was estimated to be 0.223 kg ce/kg dry matter, namely 0.818 CO_2_-eq/kg. The coefficient of fresh manure was obtained from the Lal’s (2004) review research on the carbon emission from farm operations as the lack of the study in China.

Since the lack of CF research about pesticide production in China, the coefficients of pesticide (insecticide and fungicide) used in this work were determined by West and Marland’s (2002) study in USA, including the production, packaging, transportation and application of the pesticide formulation.

Both greenhouse cover and mulching film used in all cases were plastic films, and the factor was derived from the work of Tian and Zhang (2013) on the agricultural plastic film in China. The result including the direct and indirect emission during the agricultural plastic film production in China and the carbon emission coefficient was 5.18 kg ce/kg, namely 18.993 kg CO_2_-eq/kg.

**B2 *Carbon emission factors of energy consumption***

The carbon emission coefficient of electricity was obtained from the Provincial GHG Inventory Guidelines of China (NDRC, 2011), which provide average carbon emission of power supply units in North China Regional Power Grid (including Beijing, Tianjin, Hebei, Shanxi, Shangdong and west of Inner Mongolia). Carbon emission coefficients of diesel and gasoline were calculated by multiplying the conversion factor to standard coal and the carbon emission coefficient of standard coal, which were obtained from China Energy Statistical Year Book (NBSC, 2017). The conversion factor to standard coal of diesel and gasoline were 1.4571 and 1.4714, respectively, and the conversion coefficient of carbon emissions of standard coal is 2.204 kg CO_2_-eq /kg. Therefore, the carbon emission factors of diesel and gasoline were 3.211 and 3.243 kg CO_2_-eq/kg, respectively.

**References**

Chen, S., Lu, F., Wang, X., 2015. Estimation of greenhouse gases emission factors for China's nitrogen, phosphate, and potash fertilizers (in Chinese). Acta Ecol. Sin. 35, 6371-6383.

Lal, R., 2004. Carbon emission from farm operations. Environ. Int. 30, 981-990.

National Bureau of Statistics of China (NBSC), 2017. China Energy Statistical Year Book. National Bureau of Statistics of China, Beijing.

National Development and Reform Commission of China (NDRC), 2011. Provincial GHG Inventory Guidelines (in Chinese).

Tian, Y., Zhang, J.B., 2013. Regional Differentiation Research on Net Carbon Effect of Agricultural Production in China (in Chinese). J. Nat. Resour. 28, 1298-1309.

West, T.O., Marland, G., 2002. A synthesis of carbon sequestration, carbon emissions, and net carbon flux in agriculture: comparing tillage practices in the United States. Agriculture, Ecosystems & Environment 91, 217-232.

Zhang, D., Shen, J., Zhang, F., Li, Y.e., Zhang, W., 2017. Carbon footprint of grain production in China. Scientific Reports 7, 4126.

Zhang, W.F., Dou, Z.X., He, P., Ju, X.T., Powlson, D., Chadwick, D., Norse, D., Lu, Y.L., Zhang, Y., Wu, L., Chen, X.P., Cassman, K.G., Zhang, F.S., 2013. New technologies reduce greenhouse gas emissions from nitrogenous fertilizer in China. Proc. Natl. Acad. Sci. U.S.A. 110, 8375-8380.
